# Targeting Inflammation With Nanosized Drug Delivery Platforms in Cardiovascular Diseases: Immune Cell Modulation in Atherosclerosis

**DOI:** 10.3389/fbioe.2018.00177

**Published:** 2018-11-27

**Authors:** Antonio Cervadoro, Roberto Palomba, Giuseppe Vergaro, Roberta Cecchi, Luca Menichetti, Paolo Decuzzi, Michele Emdin, Stefano Luin

**Affiliations:** ^1^NEST Laboratory, Scuola Normale Superiore, Pisa, Italy; ^2^Laboratory of Nanotechnology for Precision Medicine, Fondazione Istituto Italiano di Tecnologia, Genova, Italy; ^3^Division of Cardiology and Cardiovascular Medicine, Fondazione Toscana Gabriele Monasterio, Pisa, Italy; ^4^Institute of Life Sciences, Scuola Superiore Sant'Anna, Pisa, Italy; ^5^Center for Nanotechnology Innovation (CNI@NEST), Istituto Italiano di Tecnologia, Pisa, Italy; ^6^CNR Institute of Clinical Physiology (IFC), Pisa, Italy; ^7^NEST Laboratory, Istituto Nanoscienze, CNR, Pisa, Italy

**Keywords:** atherosclerosis, inflammatory diseases, smart nanomaterials, drug delivery, nanomedicine, imaging and theranostics, immune cells, cardiovascular diseases

## Abstract

Atherosclerosis (AS) is a disorder of large and medium-sized arteries; it consists in the formation of lipid-rich plaques in the intima and inner media, whose pathophysiology is mostly driven by inflammation. Currently available interventions and therapies for treating atherosclerosis are not always completely effective; side effects associated with treatments, mainly caused by immunodepression for anti-inflammatory molecules, limit the systemic administration of these and other drugs. Given the high degree of freedom in the design of nanoconstructs, in the last decades researchers have put high effort in the development of nanoparticles (NPs) formulations specifically designed for either drug delivery, visualization of atherosclerotic plaques, or possibly the combination of both these and other functionalities. Here we will present the state of the art of these subjects, the knowledge of which is necessary to rationally address the use of NPs for prevention, diagnosis, and/or treatment of AS. We will analyse the work that has been done on: (a) understanding the role of the immune system and inflammation in cardiovascular diseases, (b) the pathological and biochemical principles in atherosclerotic plaque formation, (c) the latest advances in the use of NPs for the recognition and treatment of cardiovascular diseases, (d) the cellular and animal models useful to study the interactions of NPs with the immune system cells.

## Atherosclerosis and inflammation

Atherosclerotic disease, or simply atherosclerosis (AS), is initially characterized by the formation of fatty streaks, with the accumulation of lipids [primarily cholesterol, but also triglycerides (Goldberg, 2018)] in the intima and inner media of arterial wall, especially in regions with abnormal flow patterns (Chistiakov et al., 2017). Fatty streaks may then evolve into soft, lipid-rich plaques, and eventually into thick cap-calcified lesions and/or unstable plaques characterized by inflammatory infiltration and sustained oxidative processes. Stable fibrocalcific atheroma is characterized by calcium deposits, small lipid deposits, slight lumen reduction, and, often, by poor functional relevance. Vulnerable atheroma, which is more prone to rupture, is characterized by a large lipid-rich necrotic core, thin fibrous cap (< 65 μm), neovascularization, spotty calcifications, inflammatory cells, and positive remodeling (Moore and Tabas, 2011; Hansson et al., 2015). The possibility of plaque regression has been reviewed in Chistiakov et al. (2017) focusing on animal models, and in Fisher (2016) also considering the dynamic changes in lipid and immune cells distributions.

AS is currently considered an inflammatory disorder, characterized by the infiltration into sub-endothelial space of various immune cells (ICs), especially circulating monocytes (MCs) that subsequently differentiate into macrophages (MΦs) and then into foam cells (FCs), going along with plaque formation and evolution (Moore et al., 2013; Zhang et al., 2017). Such simplified picture is complicated by the heterogeneity of the cells within and close to lesions during evolution [MCs, MΦs, and FCs, but also neutrophils, dendritic cells (Butcher and Galkina, 2012), T cells (Taleb, 2016), and possibly others]. In the cited reviews particular attention is given to the markers of the various IC phenotypes, although cells can present more than one function and can express a continuum of markers of different subtypes (Butcher and Galkina, 2012).

A key example regards MΦs, the characterizing cells in AS: their different subtypes can have antithetic roles. Initially classified only as classically activated M1 (pro-inflammatory) or alternatively activated M2 (anti-inflammatory), evidences have brought to the definitions of additional subtypes (e.g., Mox, M4, Mhem) and even “sub-subtypes” (e.g., M2a, M2b, M2c); there could actually be a continuum of specializations, and MΦs could even convert into each other (Butcher and Galkina, 2012; Leitinger and Schulman, 2013). It must be noted that also smooth muscle cells (SMCs) can assume some of the functions usually assigned to MΦs (efferocytosis, internalization of lipids or cholesterols), and may transform in foam cells (Chistiakov et al., 2017).

In any case, cells with phenotypes closer to Mox or M1 cells are the most active in internalizing lipids (particularly cholesterol), especially if agglomerated within oxidized low density lipoprotein (oxLDL). Upon this process, efferocytosis efficiency of cells is reduced, they transform into foam cells, and are easily subjected to imbalances in cholesterol influx/efflux process and hypoxia. The final outcome is most often apoptosis or necrosis, which resolve in local accumulation of the lipid content, the major constituent of the inner (possibly necrotic) core of the atherosclerotic plaque (Moore and Tabas, 2011). At the same time, the cell's residual components promote further inflammation signals and generation of oxidative species, in a persistent cycle of recruitment of intimal macrophages and their polarization toward pro-inflammatory subsets (Libby et al., 2014). Moreover, both monocytes and macrophages have been shown to contribute to the increase of the gamma-glutamyl transferase enzyme (γGT) in AS lesions, increasing the oxidative character of these zones (Pucci et al., 2014; Belcastro et al., 2015).

This self-sustained state of inflammation (similar to what happens in chronic wound environments) is characteristic of the vulnerable plaque. The presence of activated macrophages leads to the secretion of matrix-components degrading enzymes (such as matrix metalloproteinases-MMPs), inhibits the production of collagen by the SMCs, and finally contributes to vascular wall reshaping. These mechanisms are useful in early lesions, supporting resolution of disrupted endothelial layers and avoiding accumulation of toxic species, as well as for the reduction of atherosclerotic plaques; but, at the same time, they induce fragility in late plaques, with the possible final consequence of plaque rupture (Libby et al., 2014; Hansson et al., 2015; Martinez and White, 2018). Inflammation also modulates the clinical consequences of the thrombotic complications of AS, while its inhibition could attenuate progression, mitigate the risk of plaque rupture, and even promote regression of AS (Awan and Genest, 2015; Bäck and Hansson, 2015; Bäck et al., 2015; Kamaly et al., 2016).

## Atherosclerosis treatments

Current therapeutic approaches to AS aim at reducing promoting factors, including hypertension, smoke, and dyslipidemias; considered drugs (e.g., inhibitors of cholesterol hepatic synthesis like statins) particularly affect production and transport of cholesterol (or other lipids) to the arterial walls (Coomes et al., 2011; Duivenvoorden et al., 2014; Tsujita et al., 2015). Different approaches have been investigated, such as blocking absorption of cholesterol in the intestine (e.g., by ezetamibe) or controlling its reverse transport in ICs, e.g., by using an agonist for the liver X receptor (LXR) like GW3965, or administering artificial forms of high density lipoproteins (HDL; Duivenvoorden et al., 2014; Tsujita et al., 2015; Pulakazhi Venu et al., 2017; Goldberg, 2018; Mueller et al., 2018).

Controlling inflammation is another promising strategy; in particular, the interleukin-1 pathway has been identified as a possible therapeutic target. Canakinumab, a monoclonal antibody targeting interleukin-1β, was tested in the CANTOS (Canakinumab ANti-inflammatory Thrombosis Outcomes Study) trial (Ridker et al., 2011, 2017). Moreover, the chemotherapeutic drug methotrexate (MTX), largely used as an immunomodulatory and anti-inflammatory (AI) drug, was described to lower the risk for total cardiovascular diseases in patients with chronic inflammation (Popkova et al., 2015; Gomes et al., 2018), and has been observed to inhibit atherogenesis and macrophage migration to the intima in animal models (Bulgarelli et al., 2012). Although its mechanisms of action are not fully clear, this molecule is capable of reducing the secretion of pro-inflammatory cytokines and the expression of adhesion molecules on both immune and endothelial cells (Coomes et al., 2011).

While promising, systemic administration of AI drugs is often limited by a narrow therapeutic index and by severe adverse effects, including bone marrow suppression, neutropenia and immunodepression. Blockade and stimulation of the mechanisms involved in inflammation can hold positive, null, or even detrimental results depending on the phase of the atherogenic process (Aluganti Narasimhulu et al., 2016). Based on these premises, it is authors' belief that a rational modulation of the functions of ICs is promising for an efficient treatment and hopefully comes with fewer adverse effects.

Ideally, it would be desirable to identify an effective strategy for all of the AS phases; e.g., in (Libby et al., 2014) Resolvin E1 is indicated as a mediator that reverses all the advanced lesions associated processes, while contributing to the resolution of the plaque also in earlier phases. Another promising possibility is controlling release or activation of compounds used to alleviate or cure AS symptoms/consequences. Possible approaches include: the use of drugs modified with a glutamyl in order to exploit the increased concentration of γGT in AS lesions (Belcastro et al., 2015); developing methods exploiting high cholesterol concentrations; tackling pathways leading the transformation process from monocyte to pro-inflammatory MΦs and finally to foam cells (Rousselle et al., 2013).

Due to their ambivalent role of inducing inflammation and regulating tissue regeneration, macrophages have been the first candidates addressed in order to control AS. Vannella and Wynn presented an interesting review describing macrophages behavior in different tissues, the mediators of the various mechanisms involved, and finally several possible ways to modulate them (Vannella and Wynn, 2017). It would be ideal to force their egress from AS lesions as transformation into foam cells is occurring; to this end, a suggestive idea could be activating or releasing a “macrophage migration-enhancement factor” (Weisbart et al., 1974; Ueno et al., 1997; Nunami et al., 1998), or interfering with cell surface adhesion molecule only in over-abundancy of cholesterol or other AS markers, e.g. by stopping the phosphorylation of CD44 (Qin, 2012).

## Toward targeted theranostics NPs

As hinted above, spatial and temporal control of drug activity can reduce collateral short and long term effects; moreover, it can result in more convenient administration methods. These premises strongly call for the development of efficient, targeted delivery strategies for AI molecules, just like the ones based on their encapsulation into NPs (Costa Lima and Reis, 2015). Several kinds of NP have been considered for drug delivery; characteristics and production protocols for some of these are reviewed in Allen et al. (2016), Ulbrich et al. (2016), Cheraghi et al. (2017), and Matoba et al. (2017). There are still limitations and drawbacks for the clinical use of many NPs; these could arise from a not-yet perfect control of the final fate of many formulations, since they often accumulate also in the organs of the reticuloendothelial system (RES), from polydispersion and/or poor reproducibility in their preparation, or from the often difficult scale-up and high cost for their production, especially when multifunctional capabilities are added (Cheng et al., 2012; Ulbrich et al., 2016). However, NP physicochemical properties can be finely tailored during their synthesis and this allows optimizing drug loading and NP target specificity (Allen et al., 2016; Pentecost et al., 2016).

In the clinical setting of atherosclerotic disease, NPs loaded with anti-inflammatory drugs can be a powerful tool to hit inflammatory targets at the plaque level, preventing systemic side effects (Jokerst and Gambhir, 2011; Di Mascolo et al., 2013). Drug-loaded NPs targeting macrophages and other immune cells could control their pro-inflammatory activities and thus prevent, attenuate, and possibly reverse related disorders (i.e., increased atherosclerosis, but also altered adipocyte function and insulin resistance). At the same time, NPs could also be loaded with imaging agents allowing the detection of vulnerable atherosclerotic plaques; similar theranostic strategies already showed potential for detection and treatment of other diseases (including cancer and neurodegenerative disorders), exploiting a number of imaging modalities, among which optical imaging (OI), magnetic resonance imaging (MRI), ultrasound and photoacoustic (US-PA), computed tomography (CT), and nuclear imaging based on single photon and positron emission tomography (SPECT, PET; Xie et al., 2010; Kim et al., 2014; Weissleder et al., 2014; Atukorale et al., 2017; Stigliano et al., 2017; Zhang et al., 2017).

There is evidence that NPs can segregate into the plaque in preclinical models of AS via a “passive” targeting mechanism (Weissleder et al., 2014); while such a relative selectivity has not been completely understood, some hypotheses have been proposed. First, the abnormal hemodynamic forces where plaques develop may favor NPs deposition (Hossain et al., 2015); second, the endothelium appears discontinuous with openings for sufficiently small objects (Kim et al., 2014); third, there can be an active role of immune cells in vehiculating or accumulating the nanoparticles in inflamed districts (Moore et al., 2017).

Other strategies focused on specific targeting, e.g., toward inflammatory factors, dysfunctional endothelial cells, or specific macrophage receptors involved in cholesterol transport. Examples are drug carriers functionalization with selectin ligands or antibodies directed to CAMs (sialyl-Lewis X, PSFL-1, ICAM and VICAM ligands, anti-ICAM, anti-VCAM; Robbins et al., 2010) or anti-oxLDL receptor (Li et al., 2010). Li et al. used liposomes decorated with LOX-1 antibodies, Indium (^111^In) or Gadolinium (Gd), and DiI fluorescence markers to image atherosclerotic plaques in ApoE^−/−^ mice. Alternatively, it has been proposed to target collagen IV, which is present on the vascular basement and exposed when vascular permeability increases (Chan et al., 2011; Chen et al., 2013; Kamaly et al., 2016; Meyers et al., 2017). Also common strategies to target macrophages are based on functionalizing nanoparticles with dextran sulfate coating or peptides mimicking low-density lipoproteins (LDL) such as apolipoprotein A1 (ApoA-1), whose receptors (Class A Scavenger Receptor 1 MSR-1, and class B Scavenge Receptor CD36) are expressed on macrophages cell membrane (Canton et al., 2013). Further works focusing on visualization of macrophages distribution using nanoparticles are well reviewed in Weissleder et al. (2014).

Active targeting was achieved also using a “biomimetic” approach: NPs can derive from, or have properties similar to, aggregates like LDL or HDL, naturally accumulating in AS plaques (Allijn et al., 2013; Duivenvoorden et al., 2014; Gomes et al., 2018). More specific tissue or cell targeting can be found testing nanoparticle libraries *in-vitro* or *in-vivo* (Kamaly et al., 2016; Tang et al., 2016), but the mechanisms of the found specificity should be understood, also in order to ensure it is preserved downhill of modifications of nanoconstructs or in different biological environments. Libraries are often tested preliminarily in cell cultures, but *in-vivo* tests are necessary at least to evaluate the impact of the different biological media.

Indeed, upon entry in an organism, NPs are usually coated by a protein corona (PC), changing their biological identity. PC formation (or “opsonisation”) is often the first step toward the sequestration of NPs by the RES. Various approaches have been considered to avoid such phenomenon. Recent strategies are based on controlling nanoconstruct stiffness, since it was found that deformable particles are less subject to uptake by macrophages in RES in off-target tissues (Key et al., 2015; Palomba et al., 2018). Other methods exploit different coatings and functionalization developed for controlling the PC, e.g., by using polymers like PEG. This is thought to be an antifouling agent, but it actually seems to modulate the PC (Schöttler et al., 2016); moreover, it has been shown to be immunogenic, requiring the use of additional functionalizing moieties (Mima et al., 2017). Other possible coating molecules are based on peptides: examples comprise zwitterionic ones, for limiting serum-protein adsorption (Ranalli et al., 2017), or aptamer-likes, for enriching the PC with specific molecules present in biological fluids, which act as targeting moieties when properly oriented (Santi et al., 2017). All these approaches easily grant nanoconstructs with extended circulation half-life.

An advantage that will play important role consists in NPs potential multimodality: not only they can contain more than one drug and/or imaging agent, but it could also be possible to implement a trigger for drug release/activation. This could either be intrinsic (provided by the pathological environment) or exogenous (ultrasound, light, oscillating magnetic fields).

Especially under this view, an interesting development can arise from a synergy between nanotechnology and personalized medicine (Mendes et al., 2018). Early screening of the most suited bioactives (Tang et al., 2016; Risum et al., 2017), real time monitoring of local accumulations (Zavaleta et al., 2018), observing early feedbacks to the treatment (Qiao et al., 2017), and predicting patient responses (Sykes et al., 2016) are all possible aspects of personalized medicine. We believe that the rational application of nanotools in all these steps will embody a fundamental role in the upcoming clinical and pre-clinical research (Mura and Couvreur, 2012; Polyak and Ross, 2017; Yordanova et al., 2017).

## NPs for AS treatment and diagnosis

The use of NPs for treatment of AS and visualization of plaques up to 2015 has been elegantly reviewed in Zhang et al. (2017); we further selected more recent works not reported there (Table 1). In this session, we describe some of these and other relevant works.

**Table 1:** Selected recent original works on detection and treatment of atherosclerosis using nanoparticles.

**Molecular/functional target**	**Nanoparticles**	**Imaging platforms**	**Animal model/patients, dose, and administration route**	**Results**	**References**
Macrophages; TNF-α, MMP9.	LDE: Lipid core NPs resembling the lipid structure of low-density lipoprotein, carrying PTX and/or MTX.	*Ex-vivo* optical imaging.	*Model:* 38 New Zealand white rabbit, 1% cholesterol diet for 8 weeks. *- Treatment:* I.V. injections 4 mg/kg 1/w.	Increased regression of plaque areas (−59%) and of intima area (−57%) by LDE-PTX + LDE-MTX. Macrophage presence in aortic lesions reduced (−48% by LDE-PTX, −43% by LDE-PTX + LDE-MTX). Reduced expression of MMP-9 (−74% LDE-PTX, −78% LDE-PTX + LDE-MTX) and TNF-α (-65% by LDE-PTX, −79% by LDE-PTX + LDE-MTX).	Gomes et al., 2018
Macrophages, foam cells.	Lipid coated polymeric NPs loaded with MTX.	Fluorescence imaging, PET/CT.	*Model:* ApoE^−/−^ male mice on HFD. *- Treatment:* retro-orbital NPs injection, with 20 μg equivalent of MTX, 2/w, 30 days.	Fifty percent less plaque coverage (athero-protective effect) in the aortic arch as compared to the control groups of saline and free MTX injection (*p* < 0.05).	Stigliano et al., 2017
Monocytes and macrophages, reverse cholesterol efflux.	Library of high-density lipoprotein-mimicking NPs loaded with liver X receptor agonist GW3965.	Fluorescence imaging, PET, NIRF.	*Model:* ApoE^−/−^ male mice. *- Treatment:* 10 mg/kg equivalent of GW3965, I.V. 2/w, 6 weeks.	Rational library screening strategy for identifying NPs with favorable immune cell specificity and biodistribution in an AS mouse model. 28% reduction of total lipids in aortic macrophages, 43% reduction in monocytes, 40% reduction in all CD45^−^ non-immune cells. Abolished GW3965 liver toxicity.	Tang et al., 2016
Collagen IV.	Col-IV IL-10 NP22: polymeric NPs containing anti-inflammatory IL-10 and decorated with the targeting peptide *Col IV*.	Confocal fluorescence microscopy.	*Model:* Ldlr^−/−^ mice fed a Western-type high-fat/high-cholesterol diet for 12 weeks. *- Treatment:* Col IV Il-10 NPs (5 μg /mouse equivalent of IL-10) I.V. 1/w for 4 weeks.	In lesions: oxidative stress significantly decreased compared with control NPs; no effects on macrophage or smooth muscle cell content. Stabilized and remodeled pre-existing advanced atherosclerotic plaques, via improved fibrous cap thickness and decreased necrotic core.	Kamaly et al., 2016
Monocytes, macrophages.	Polymer based spherical NPs loaded with pioglitazone and/or fluorescein isothiocyanate.	Flow cytometry, *ex-vivo* optical microscopy.	*Model:* ApoE^−/−^ mice under HFD. 1.9 mg/kg/day angiotensin II administered intraperitoneally. *- Treatment:* Weekly treated with injection of pioglitazone-NP (7 or 0.7 mg/kg/week)	Altered inflammatory polarity of peripheral monocytes; tissue macrophage polarity regulated toward less inflammatory phenotypes (M2) by suppressing the EMMPRIN/MMP pathway; atherosclerotic plaque destabilization and rupture more effectively inhibited than with oral pioglitazone. NPs found in circulating monocytes and aortic macrophages.	Nakashiro et al., 2016
Macrophages, atherosclerotic plaque.	Hyaluronan NPs (HA-NPs).	Super Resolution Microscopy (dSTORM), PET/MRI.	*Models:* ApoE^−/−^ mice with early (6 weeks HFD) or advanced (12 weeks HFD) AS; New Zealand white male rabbits with angioplastical endothelial denudation of the aorta. *- Treatment:* 25 mg/kg (for biodistribution) or 50 mg/kg/week (therapeutic study) of HA equivalent.	HA-NPs uptake by macrophages in early aortic lesions is five-fold higher than in advanced lesions. Lesions were significantly smaller than in control groups, with 30% fewer macrophages, and 30–40% higher collagen content, an important factor for plaque stability. No relevant relative differences between the organs bio-distributions between mice and rabbits, underlining the translational aspect of the study.	Beldman et al., 2017
Collagen IV, arterial injury.	Gold NPs coated with a collagen-binding peptide labeled with Alexa Fluor 546.	Fluorescence microscopy.	*Model:* adult male Sprague–Dawley rats after carotid artery balloon injury. *- Treatment:* NPs I.V. injection (2 mg/kg).	Fluorescence was detected at the site of left carotid arterial injury; no fluorescence was detected for controls conditions. Fluorescence can be detected from 20 min up to 96 h post injection, and the fluorescence pattern shifts from binding within the arterial media toward adventitial binding.	Meyers et al., 2017
EMMPRIN.	NAP9: Paramagnetic-fluorescent micellar NPs conjugated with the EMMPRIN binding peptide AP-9.	Echocardiography, MRI, confocal fluorescence microscopy.	*Model:* Wild type C57BL/6 mice with ischemia induced by coronary artery ligation. *- Treatment:* Single injection 50 mg/kg, I.V.	The research focused on acute myocardial infarction, but EMMPRIN has an active role also in AS. Treatment resulted in improved heart contractility, reduced cardiac necrosis, and reduction of levels of MMP-2 and MMP-9 almost to those in healthy animals.	Cuadrado et al., 2016

*AS, atherosclerosis; PET, positron emission tomography; CT, computed tomography; PTX, Paclitaxel; MTX, Methotrexate; I.V., intravenous; NP, nanoparticle; MMP, matrix metalloproteinase; TNF, tumor necrosis factor; HFD, high fat diet; ApoE, apolipoprotein E; w, week(s); NIRF, near infrared fluorescence; IL, interleukin; EMMPRIN, extracellular matrix metalloproteinase inducer; Ldlr, low density lipoprotein receptor*.

Often, intrinsic properties of NPs lead to the exploitation of drug delivery strategies together with photothermal- and radiofrequency-mediated triggering effects; e.g., Johnston proposed the possibility to use photothermal destruction of macrophages using iron oxide NPs with thin gold and dextran coating, excited by a laser pulse at 755 nm. In this manuscript the particles were used for MRI and *in vitro* photothermolysis (Ma et al., 2009). A similar approach has been tested in the NANOM-FIM trial (Kharlamov et al., 2015); here, NPs composed by silica shells containing gold and eventually magnetic nanoparticles were delivered on AS plaques by a bioengineered on-artery patch or using a magnetic navigation system; detonation of NPs using a NIR laser caused a significant final reduction of the total atheroma volume.

A different, interesting strategy consists on preventively acting on the selective recruitment of monocytes from the precursors of pro-inflammatory macrophages M1 (Nakashiro et al., 2016; Matoba et al., 2017). The authors proposed polymeric NPs loaded with Pioglitazone, an agonist of the receptor PPARγ shown to be able to influence macrophage polarization. The formulation was tested in ApoE^−/−^ mice fed a high fat diet (HFD) and infused with angiotensin II, promoting inflammation driven by monocytes/macrophages. 2 days post injection the ratio between peripheral pro- and non-inflammatory monocytes subsets decreased substantially, and tissue macrophage polarity was regulated toward the non-inflammatory phenotype M2, with consequent suppression of EMMPRIN/MMP pathway and reduction of plaque destabilization risk.

In a similar approach, Stigliano et al. (2017) confirmed the potentiality of MTX in preventive-oriented treatments by loading it into NPs. The authors demonstrated specific accumulation of NPs into macrophages residing within lipid-rich plaques along the arterial tree. In the aortic arch of ApoE^−/−^ mice on HFD treated with MTX-NPs, 50% less coverage of plaques was found in comparison to the control group. Importantly, this result was obtained by injecting a dose four times lower than reported in literature (Leite et al., 2015; Gomes et al., 2018).

MTX has also been investigated in synergy with different bioactive compounds. The group of Serrano Jr. (Gomes et al., 2018) tested the combined effect of injecting Paclitaxel (PTX)-loaded LDL-mimicking NPs and MTX-loaded NPs. In New Zealand white rabbits on atherogenic diet treated with both the NPs formulations, the regression of the lesion area and the intimal width reduction corresponded to 17 and 63%, respectively, compared to the group treated with PTX-NPs alone. The authors speculate that this athero-regression is mostly due to the macrophage reduction effect and not to an inhibition of SMCs migration into the intima.

Other possible applications of nanomedicine in the treatment of AS are based on gene regulation. Majmudar et al injected siRNA-containing polymeric NPs to silence the expression of C–C chemokine receptor type 2 (CCR-2), a key player in recruiting inflamed monocytes to atherosclerotic plaques; they observed reduced PET signals from ^89^Zr-labeled dextran nanoparticles in aortic root when compared to mice treated with an irrelevant siRNA (Majmudar et al., 2013). Another example is the downregulation of the tissue inhibitor of metalloproteinase 3 by the use of miR-712, delivered by cationic lipid NPs targeting VCAM1. The treatment was performed in ApoE^−/−^ mice and was able to significantly attenuate the development of atherosclerotic plaques (Kheirolomoom et al., 2015).

## Tools and ideas for additional research

As reviewed above, fundamental and pre-clinical researches on AI-drugs-loaded NPs against AS are beginning to bloom, and there are also clinical trials for the (separated) use of NPs and AI drugs for regression or consolidation of AS plaques. More fundamental and translational research on the mechanisms underlying their action could inspire and motivate future more efficient clinical trials using AI-drug loaded NPs against AS.

Important future steps for the development of new NP-based theranostics strategies can arise from the study of interaction mechanisms between NPs and immune cells, with particular regard to what fate NPs and their components will face upon site deposition and cellular uptake. Investigations on these directions will remarkably improve the development of NPs rational designs aimed at specific accumulation and cargo smart activation.

Indeed, where, how and in which proportion NPs are internalized in the various subtypes of ICs is still not clear, also due to the different internalization routes observed even in close phenotypes (Lunov et al., 2011), nor it is clear how or if they are exocytosed (Oh and Park, 2014). In particular, are NPs mostly internalized by circulating monocytes that enter the lesions, or by resident macrophages in lesions, where NPs enter because of the enhanced permeability of inflamed endothelium?

Immortalized cell lines can be used for preliminary experiments. The most used are murine macrophage-like RAW 264.7 and J774, and human monocyte-like THP-1 and U937 (Luster et al., 1995; Qin, 2012; Andreu et al., 2017) reviews in particular the use of THP-1, unchanged or differentiated toward a MΦ phenotype (especially M1), but also cites other human monocyte/macrophage models. Following these models, even though there are evident parallelisms, care must be taken for intrinsic differences: between cells originating from different organisms (Ingersoll et al., 2010; Matoba et al., 2017; Zhang et al., 2017); between immortalized cell lines and primary cells (Andreu et al., 2017); even between possible different differentiations ICs may undergo during regular cultures. Primary cells should be used in final tests, but in this case the last type of unwanted differentiations are even more critical [e.g., monocytes are extremely sensible and may be activated just by sole adhering on surfaces (Belcastro et al., 2015)]. For these reasons, a thoughtful characterization of their phenotypes (by visual inspection or, better, by markers analysis) should be carried out before every experiment.

Together with the internalization pathway, also the bio-distribution of NPs deserves attention; a nice review for both these issues, which considers MC and MΦs, can be found in Pentecost et al. (2016), with interesting discussions about dependencies on NP geometry and surface chemistry, targeting, control on MΦ phenotype, and imaging. In addition, medium and long-term fates of nano-construct components (in particular its bioactive cargo) should be addressed; these details can indeed impact the efficacy of the researched treatment, but also the long-term effects on the patients' health.

Finally, various animal models have been developed for the different phases of AS, as reviewed in Emini Veseli et al. (2017) and Lee et al. (2017). A recently developed model of controllable and reversible hypercholesterolemia is based on transient knockdown of the hepatic LDL receptor (LDLR) by antisense oligonucleotides in wild type C57BL/6 mice, followed by its rapid restoration (Basu et al., 2018).

## Conclusion

Cardiovascular events, such as acute myocardial infarction and stroke, are often associated with erosion/rupture of arterial atherosclerotic plaques and superimposed thrombosis. These can cause arterial-vessel occlusion, downstream ischemia and necrosis, with subsequent heart failure and the whole clinical spectrum of vascular encephalopathies. Being AS an important source of morbidity and mortality, intense research efforts toward precision and personalized medicine in this field are motivated.

There are several reviews for selected aspects of the highly complicated issue of AS and its theranostics with NPs (Table 2). Here, we shortly presented the broad background necessary to understand the use of NPs for smart delivery and activation of anti-inflammatory drugs. We tried to focus on how basic science, together with the translational and clinical aspects, can address different portions of this, aiming at providing a better answer for the underlying mechanisms of NPs intra-plaque internalization, as well as for the molecular and cellular processes at the bases of different treatments. We stressed that a more thorough understanding of these would help foresee possible side effects, even long term ones, and also the opportunity to employ similar techniques and (nano)tools to other pathologies.

**Table 2:** Selected reviews.

**Review focus**	**Covered topics**	**Covered period**	**Study models treated**	**Remarks**	**References**
Nanoparticle mediated detection and treatment of AS; prevention of plaque progression.	Atherogenesis, NPs for structural/functional imaging and therapy, preclinical stages, anti-inflammation and lipid lowering strategies, targeting routes.	Mainly 2000–2015, back to 1993.	*In vitro, in vivo*, pre-clinical stage.	NPs efficiency in the field is plenty documented *in vitro* and *in vivo* only, preclinical and clinical studies still fall behind. Useful tables summarizing researches done on detection and treatment of atherosclerosis using nanoparticles.	Zhang et al., 2017
Immune cells in healthy and AS-prone aorta.	(Sub)phenotypes classification of dendritic cells and MΦs possibly present in aortic walls, their roles in AS; mixed phenotypes	Mainly 2004–2011, back to 1913.	*In vitro, in vivo*.	Useful table of ICs classification, markers, secreted factors, functions, impact on plaque stability.	Butcher and Galkina, 2012
Lipids in cardiovascular systems	Metabolism and blood transport of cholesterols, triglycerides and other lipids.	1973–2017, one from 1954.	*In vitro, in vivo, clinical*.	Focus on the role of triglycerides, but contains a short and efficient discussion on cholesterol metabolism and transport, interaction with MΦs and role in AS.
NPs as drug delivery systems for cardiovascular disorder.	NPs performances on drug kinetics and toxicity, various nanoconstructs design/synthesis and their physiological behavior, AS, most fundamental MCs and MΦs types in humans and mice.	Mainly 2005–2016, back to 1994.	*In vitro, in vivo*, pre-clinical stage.	Short review.	Matoba et al., 2017
Vascular targeting of NPs for molecular imaging of diseased endothelium.	Rational design of physicochemical NPs properties, AS, cancer, disease-impaired blood flows. Imaging modalities (nuclear, optical, computed, tomography, magnetic resonance, ultrasound, multimodal) for NPs.	Mainly 2006–2016, back to 1981.	*In vitro, in vivo*, pre-clinical stage.	Imaging should be pondered to achieve complex information, not just images. No significant discrepancies in clinical translation of results from animal models of the disease. Authors feel a lack in clear clinical relevance or of a well-defined endpoint; this leads to poorly designed preclinical studies. However, also early-stage researches in the field are starting to consider clinical deployments.	Atukorale et al., 2017
Rational design of NPs for AS.	Early and late stage of the disease, NPs/cell interaction, biodistribution, drug delivery, multi-modal imaging, AS-oriented gene therapies.	Mainly 2008–2016, back to 1958.	*In vitro, in vivo*, pre-clinical stage.	Useful table with a summary of nanomaterials designed to image or modulate atherosclerotic lesions.	Allen et al., 2016
Taking advance of the immune system cells for therapeutic purposes.	NPs fate upon injection, physicochemical properties for rational design, cellular uptake mechanism, passive and active targeting. MΦs subtypes, markers, produced cytokines, polarization control; imaging of inflammation.	Mainly 2003–2015, back to 1977.	*In vitro, in vivo*, clinical.	NPs targeting MΦs and controlling their polarization are just beginning to be elaborated by the community and probably their potential are not yet grasped. Authors suggest the implementation of MΦs specificity in theranostic nano-constructs constitutes a strategic step to expedite transition into clinical phase.	Pentecost et al., 2016
Nanoliposomes and NPs toward cardiovascular related disorders.	Principles of action, drug release and interaction, infarcted heart, lesions imaging modalities.	Mainly 2005–2016, back to 1998.	*In vitro, in vivo*, clinical.	Nanoliposomal formulations are promising vectors for cardiovascular disorders diagnosis and treatment, but side effects should be reduced bioactives controlled release optimized, also for fastening clinical translation.	Cheraghi et al., 2017
Mouse models for AS.	Disease development and plaque rupture, pro and cons of most common mouse models, endoplasmic reticulum stress, mitochondrial dysfunction.	Mainly 2004–2016, back to 1977.	*In vivo*, clinical.	The complexity of the topic does not allow for a single, good-for-all animal model. Importantly, a correct study design must go through the identification and comprehension of the molecular events involved.	Lee et al., 2017
Animal models for AS.	Mice models for AS and for plaque rupture, rabbit models, pigs, non-human primates.	Mainly 2000–2016, back to 1980.	*In vivo*.	One discriminant among different animal models for AS plaques is the topography of the generated lesions, compared to humans. In this scenario mice became the predominant species for models related to inflammatory cardiovascular diseases. Rabbits fall just behind and are mainly exploited due to their plasma metabolism similarity with humans. ApoE^−/−^Fbn1^C1039G+/−^ mice hold high promises for the future, being the very first animal model presenting spontaneous plaque rupture with end-points similar to humans.	Emini Veseli et al., 2017

*AS, atherosclerosis; IC, immune cell; MC, monocyte; MΦ, macrophage; NP, nanoparticle; ApoE, apolipoprotein E*.

While single studies can be carried out in specialized groups, a deeper understanding of concepts, mechanisms and applicability of the developed (nano)tools needs a highly interdisciplinary environment, involving e.g. cardiologists, biologists, physicists, chemists, engineers; professionals able to talk with each other, and fostering the formation of multi-faceted scientists.

## Author contributions

SL conceived the idea. SL and AC wrote the manuscript, with major contributions from RP, RC, and GV, starting from texts written in collaboration with LM, ME, and PD. All authors discussed, edited, and contributed to the manuscript.

### Conflict of interest statement

The authors declare that the research was conducted in the absence of any commercial or financial relationships that could be construed as a potential conflict of interest.
